# Antipyretic efficacy and tolerability of oral ibuprofen, oral dipyrone and intramuscular dipyrone in children: a randomized controlled trial

**DOI:** 10.1590/S1516-31802006000300005

**Published:** 2006-05-04

**Authors:** Judith Prado, Raúl Daza, Oscar Chumbes, Iván Loayza, Luis Huicho

**Keywords:** Antipyretics, Dipyrone, Ibuprofen, Randomized controlled trial [Publication Type], Fever, Antipiréticos, Dipirona, Ibuprofeno, Ensayo controlado aleatorio [tipo de publicacion], Fiebre

## Abstract

**CONTEXT AND OBJECTIVE:**

Dipyrone is a widely used over-the-counter antipyretic in Latin America, and elsewhere among Latin immigrants. Despite limited evidence, physicians often prescribe oral ibuprofen or intramuscular dipyrone as the most effective antipyretics. Our aim was to compare the antipyretic efficacy and tolerability of a single dose of oral ibuprofen, oral dipyrone or intramuscular dipyrone in febrile children.

**DESIGN AND SETTING:**

Randomized, single-blind clinical trial, at San Bartolomé Mother-Child National Teaching Hospital, Lima, Peru.

**METHODS:**

Children from six months to six years old with fever (rectal temperature: 38.3 to 39.8° C) in the emergency ward between February and June 2003 were eligible. Seventy-five children were randomly assigned to receive a single dose of oral ibuprofen (10 mg/kg), oral dipyrone (15 mg/kg) or intramuscular dipyrone (15 mg/kg). The primary outcome was mean temperature reduction after 30, 45, 60, 90 and 120 minutes. Secondary outcomes were fever-associated symptoms and clinical adverse events.

**RESULTS:**

Fever decreased by about 0.5° C after 45 minutes and by about 1.0° C after 120 minutes in all three groups. Mean temperatures were similar for the three groups at all times. There was a significant decrease in fever-associated symptoms for all groups. Six patients (four receiving oral dipyrone and two receiving ibuprofen) were withdrawn because of vomiting within 20 minutes after first dose of study medication. One patient assigned to oral ibuprofen presented transient urticaria.

**CONCLUSIONS:**

Antipyretic efficacy and tolerability were similar for oral ibuprofen, oral dipyrone and intramuscular dipyrone. Oral antipyretics seem more appropriate for feverish children.

## RESUMO

**CONTEXTO Y OBJETIVO:**

La dipirona es ampliamente usada como medicamento de venta libre en América Latina, en los Estados Unidos de Norteamérica y en Europa, particularmente por inmigrantes de origen latino. A pesar de una evidencia limitada, los médicos precriben a menudo ibuprofeno oral o dipirona intramuscular como los antipiréticos más efectivos. El objetivo del estudio es comparar la eficacia anti-pirética y la tolerabilidad de una dosis única de ibuprofeno oral, dipirona oral y dipirona intramuscular en niños febriles.

**TIPO DE ESTUDIO Y LUGAR:**

Ensayo clínico aleatorio simple ciego realizado en el Hospital Nacional Docente Madre-Niño San Bartolomé, Lima, Perú.

**MÉTODOS:**

Fueron elegibles niños de seis meses a seis años de edad con fiebre (definida como temperatura rectal de 38.3° C o más) que acudieron a la unidad de emergencia entre Febrero y Junio del 2003. Setenta y cinco niños fueron asignados al azar para recibir una dosis única de ibuprofeno oral (10 mg/kg), dipirona oral (15 mg/kg), o dipirona intramuscular (15 mg/kg). El punto final primario fue la reducción promedio de la temperatura a los 30, 45, 60, 90 y 120 minutos. Los puntos finales secundarios fueron síntomas asociados a la fiebre y eventos clínicos adversos.

**RESULTADOS:**

La fiebre declinó en aproximadamente 0.5° C a los 45 minutos y en aproximadamente 1.0° C a los 120 minutos en los tres grupos. La temperatura promedio fue similar en los tres grupos en todos los periodos de evaluación. Hubo una reducción significativa de los síntomas asociados a fiebre en los tres grupos. Seis pacientes (cuatro del grupo dipirona oral y dos del grupo ibuprofeno oral) fueron retirados del estudio por vómitos que ocurrieron dentro de los 20 minutos luego de la primera dosis de la medicación de estudio. Un paciente del grupo ibuprofeno oral presentó una urticaria transitoria.

**CONCLUSIONES:**

La eficacia antipirética y la tolerabilidad fueron similares para ibuprofeno oral, dipirona oral y para dipirona intramuscular. Los antipiréticos orales parecen más apropiados en niños febriles.

## INTRODUCTION

Fever is a frequent manifestation of diseases in children and accounts for a substantial proportion of pediatric emergency consultations. Previous studies have shown differing results regarding the antipyretic efficacy and safety of paracetamol and ibuprofen.^1–3^ Two recent systematic reviews concluded that there was insufficient evidence regarding the superiority of paracetamol compared with placebo or physical methods and that the data available on adverse events were limited.^4,5^

There are few studies comparing the antipyretic efficacy of dipyrone with other commonly used antipyretics. The results are conflicting.^6–9^ Methodological problems in these studies have precluded a definite conclusion.

Dipyrone is a widely used antipyretic agent in developing countries, particularly in Latin America, and in the United States and Europe, most frequently among Latin immigrants.^10^ It is commonly available as an over-the-counter drug in several countries, including Peru. Oral ibuprofen and intramuscular dipyrone are frequently used by local physicians on the assumption that they are the most effective antipyretics.

In addition, intramuscular dipyrone is frequently demanded by parents and prescribed by physicians at the emergency wards, in the belief that it is faster than oral antipyretics for abating fever in children. Thus, we were prompted to compare the antipyretic efficacy of a single dose of oral ibuprofen, oral dipyrone and intramuscular dipyrone in febrile children.

## OBJECTIVE

To compare the antipyretic efficacy and tolerability of a single dose of oral ibuprofen, oral dipyrone and intramuscular dipyrone in febrile children.

## METHODS

Children aged six months to six years old in the emergency ward of San Bartolomé Mother-Child National Teaching Hospital, Lima, Peru, were eligible. The study period was from February to June 2003. Children were included if they had a rectal temperature of between 38.3 and 39.8° C and were able to stay in the emergency ward for at least two hours. A complete clinical assessment, including past and current medical history, was performed. Children with any of the following criteria were excluded: antipyretic treatment within the last four hours, history of allergy to any of the study medications, severely ill children, history of seizures, frequent vomiting within the previous two hours, history of liver, renal or hematological disease, immune suppression, or severe malnutrition (by weight-for-age assessment).

The study protocol was approved by the institutional ethics committee and the San Marcos University Postgraduate Unit, Lima, Peru. Before any study procedure was carried out, an informed consent form was signed by the parents or legal guardians of the eligible children.

Patients were randomly assigned to oral ibuprofen (10 mg/kg), oral dipyrone (15 mg/kg) or intramuscular dipyrone (15 mg/kg) by means of a random numbers table. The study medication was administered by staff nurses and, thus, the investigators were blinded. Similarly, the randomization codes were not known to the investigators and were kept separately until the end of the study.

**Figure 1:**
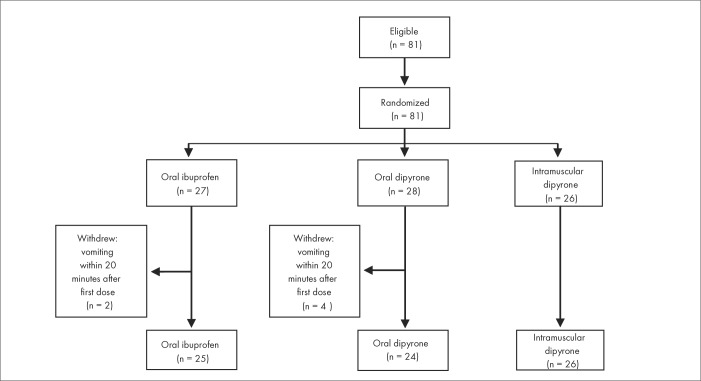
Summary profile of the study of treatment of fever in children.

The primary outcome was mean temperature reduction after 30, 45, 60, 90 and 120 minutes. The secondary outcomes were fever-associated symptoms and clinical adverse events. Concomitant therapy, administered at the discretion of the physician in charge, included systemic corticosteroids, nebulized beta-adrenergics and oral rehydrating solution. Any additional laboratory test or procedure was ordered at the discretion of the physician in charge, without the investigators’ participation or influence. A summary profile of the study is shown in Figure 1.

Rectal temperature was taken before the first dose of the study medication and then after the study times of 30, 45, 60, 90 and 120 minutes. A mercury thermometer was used, introducing the bulb 2 cm inside the rectum for a five-minute period. In addition to rectal temperature measurement, the parents or legal guardians were asked about their child's accompanying clinical symptoms, such as crying, irritability, anorexia, hypoactivity, shivering and vomiting, upon entry and then at every subsequent assessment. These data were recorded as present or absent.

Ibuprofen in oral suspension (Afebril^®^) was provided by the local representative of Roemmers, and dipyrone (Antalgina^®^) by Sanitas, Lima, Peru. The pharmaceutical laboratories had no role in study design, data collection, data analysis, data interpretation, or writing of the report.

Immersion in water at 32 to 36° C (neutral temperature), as measured via a water thermometer, was applied for 15 minutes to all children, within five minutes after the first dose of the study drug had been administered.

Whenever the temperature had not decreased by the time 120 minutes was reached, or if it was found to have increased by 0.5° C at any assessment time, an additional dose of the assigned medication was administered.

The sample size was calculated on the basis of 90% statistical power and an error margin of 0.05. The chi-squared test and analysis of variance were performed for categorical and continuous variables, respectively, by means of the Statistical Package for the Social Sciences (SPSS) 11.0 statistical software.

## RESULTS

Baseline demographic and clinical characteristics did not differ between the three groups (Table 1). Most patients were managed on an outpatient basis. None of the children assigned to oral ibuprofen were hospitalized, whereas one on oral dipyrone and two on intramuscular dipyrone were subsequently hospitalized.

**Table 1.** Baseline clinical and demographic characteristics of febrile children and treatments for fever*

Variable	Oral ibuprofen (n = 25)	Oral dipyrone (n = 24)	Intramuscular dipyrone (n = 26)
**Female (%)**	13 (52)	13 (54)	13 (50)
**Mean age in months (SD)**	17.9 (12.0)	16.3(13.7)	21.0 (18.3)
**Weight in kg (SD)**	10.8 (2.9)	10.1 (2.4)	10.6 (3.5)
**Mean baseline temperature (°C) (SD)**	39.0 (0.5)	38.8 (0.4)	38.9 (0.5)
**Main diagnosis**			
**Upper respiratory tract infection (%)**	16 (64)	15 (63)	16 (61)
**Lower respiratory tract infection (%)**	1 (4)	1 (4)	1 (4)
**Gastroenteritis (%)**	6 (24)	7 (29)	7 (27)
**Urinary tract infection (%)**	1(4)	0	0
**Other condition (%)**	1 (4)	1 (4)	2 (8)
**Concomitant clinical condition**			
**Bronchial obstruction (%)**	10 (40)	6 (25)	8 (31)
**Down syndrome (%)**	0	2 (8)	0
**Others (%)**	2 (8)	1 (4)	2 (8)
**Concomitant therapy**			
**Systemic corticosteroids (%)**	7 (28)	4 (17)	3 (12)
**Beta-adrenergic (%)**	7 (28)	3 (13)	3 (12)
**Oral rehydrating solution (%)**	4 (16)	4 (17)	5 (19)
**Nutritional status (weight for age)**			
**Well-nourished (%)**	19 (76)	23 (96)	20 (77)
**Malnourished (%)**	6 (24)	1 (4)	6 (23)

* *Differences between groups were not statistically significant. SD = standard deviation.*

**Figure 2:**
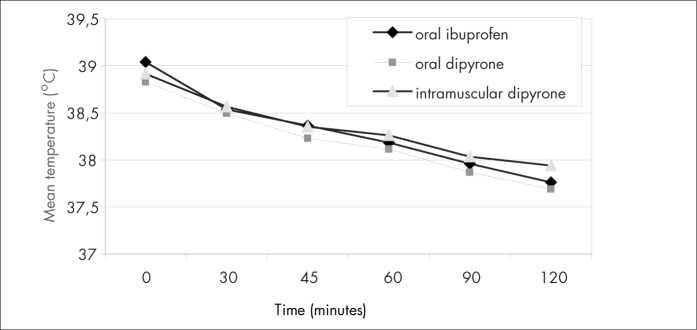
Mean temperature at different times.

**Figure 3:**
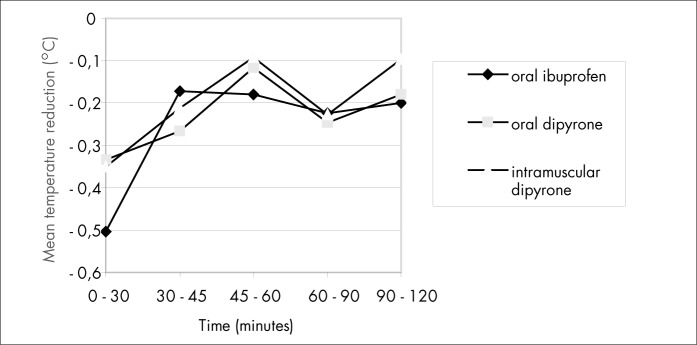
Mean temperature variation between measurement times.

**Figure 4:**
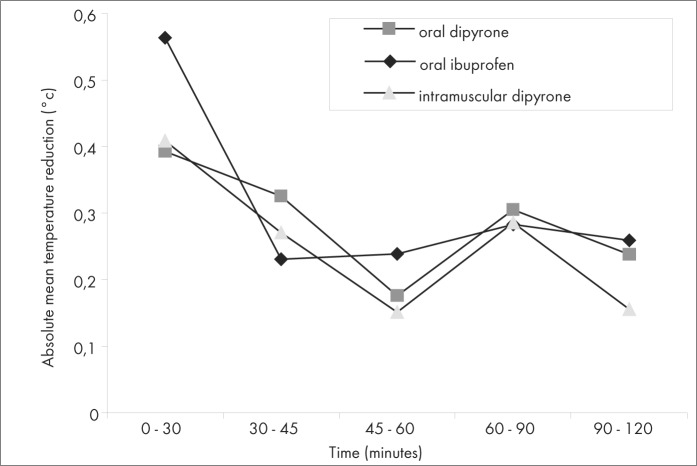
Absolute mean temperature reduction between measurement times.

The mean temperatures in the three groups did not differ at the times of 45, 60, 90 and 120 minutes (Table 2 and Figure 2). Although the oral ibuprofen group showed a significantly lower mean temperature at 30 minutes, this difference was clinically irrelevant. The mean temperature reduction from time zero to 120 minutes was not significantly different between the three groups at any moment (Table 3, Figure 3 and Figure 4). A moderate and relatively rapid reduction of fever was demonstrated for all three drugs. That is, the fever decreased by about 0.5° C after 45 minutes and by about 1.0° C after 120 minutes in all three groups.

**Table 2.** Mean (± SD) rectal temperature values (in °C) for the three study groups of febrile children at different times

Time (minutes)	Oral ibuprofen (n = 25)	Oral dipyrone (n = 24)	Intramuscular dipyrone (n = 26)	95% CI	p
**0**	39.04 ± 0.51	38.83 ± 0.42	38.92 ± 0.47	38.82 - 39.04	0.294
**30**	38.54 ± 0.63	38.49 ± 0.39	38.57 ± 0.58	38.41 - 38.66	0.03*
**45**	38.36 ± 0.51	38.23 ± 0.45	38.35 ± 0.58	38.19 - 38.44	0.602
**60**	38.18 ± 0.47	38.11 ± 0.45	38.26 ± 0.53	38.08 - 38.29	0.559
**90**	37.96 ± 0.38	37.87 ± 0.46	38.03 ± 0.49	37.85 - 38.06	0.418
**120**	37.76 ± 0.41	37.69 ± 0.37	37.94 ± 0.49	37.69 - 37.89	0.107

* *Sfafisfically significant; SD = Standard deviation; CI = 95% confidence interval.*

**Table 3.** Mean (± SD) temperature variation (in °C) between measurement times for the three study groups of febrile children

Time (minutes)	Oral ibuprofen (n = 25)	Oral dipyrone (n = 24)	Intramuscular dipyrone (n = 26)	95% CI	P
**0 - 30**	- 0.50 ± 0.27	- 0.33 ± 0.36	- 0.35 ± 0.34	0.3201 - 0.4719	0.132
**30 - 45**	- 0.17 ± 0.32	- 0.27 ± 0.17	- 0.21 ± 0.22	0.1595 - 0.2725	0.405
**45 - 60**	- 0.18 ± 0.24	- 0.12 ± 0.20	- 0.09 ± 0.20	0.0796 - 0.1791	0.334
**60 - 90**	- 0.22 ± 0.29	- 0.25 ± 0.39	- 0.23 ± 0.23	0.1623 - 0.3017	0.964
**90 - 120**	- 0.20 ± 0.30	- 0.18 ± 0.24	- 0.10 ± 0.27	0.0945 - 0.2202	0.361

*SD = standard deviation; CI = confidence interval.*

The frequencies of crying, anorexia, hypoactivity, shivering and vomiting were similar for all three groups (Table 4). Also, there was a significant decrease in the fever-associated symptoms for all three groups, which was concurrent with the decrease in fever (Table 4).

**Table 4.** Tolerability outcomes for three different treatments for fever in children at different times*

Clinical sign present	Time (minutes)	Oral ibuprofen n (%)	Oral dipyrone n (%)	Intramuscular dipyrone n (%)
**Irritability**	***0***	14 (56)	16 (67)	17 (65)
	***30***	9 (36)	11 (46)	12 (46)
	***45***	2 (8)	8 (33)	8 (31)
	***60***	0	5 (21)	3 (12)
	***90***	0	1 (4)	1 (4)
	***120***	0	0	1 (4)
**Crying**	***0***	13 (52)	11 (46)	15 (58)
	***30***	6 (24)	8 (33)	8 (31)
	***45***	3 (12)	6 (25)	6 (23)
	***60***	1 (4)	4 (17)	4 (15)
	***90***	0	0	3 (12)
	***120***	0	0	2 (8)
**Anorexia**	***0***	6 (24)	4 (17)	5 (19)
	***30***	0	1 (4)	1 (4)
	***45***	0	1 (4)	0
	***60***	0	0	0
	***90***	0	0	0
	***120***	0	0	0
**Hypoactivity**	***0***	3 (12)	5 (21)	4 (15)
	***30***	0	1 (4)	0
	***45***	0	0	0
	***60***	0	0	0
	***90***	0	0	0
	***120***	0	0	0
**Chilling**	***0***	1 (4)	0	3 (12)
	***30***	0	0	0
	***45***	0	0	0
	***60***	0	0	0
	***90***	0	0	0
	***120***	0	0	0
**Vomiting**	***0***	0	0	0
	***30***	0	0	0
	***45***	0	0	0
	***60***	0	0	0
	***90***	0	0	0
	***120***	0	0	0

* *differences between groups were not statistically significant.*

Six patients (four from the oral dipyrone group and two from the ibuprofen group) were withdrawn from the study because of vomiting within 20 minutes after receiving the first dose of antipyretic intake. There was only one case of mild, transient urticaria, which appeared 30 minutes after oral ibuprofen administration in a girl aged 9.1 months. She had a previous history of allergic rhinitis that had been treated with cetirizine seven days before enrolment. The urticaria remitted by the time of reaching three hours after ibuprofen administration, without any specific therapy.

## DISCUSSION

Our results showed similar antipyretic effects from oral ibuprofen, oral dipyrone and intramuscular dipyrone. Also, the rate of adverse effects was similar for the three study medications.

Since physical methods are commonly used in the initial management of fever in children, we applied water immersion to all the patients included in this study. On the basis of previous reports,^11^ we assumed that the temperature reduction due to drug administration was not significantly affected by this potentially confounding factor.

Our study also showed that intramuscular dipyrone was not faster than oral ibuprofen in abating fever, and that safety and tolerability were similar for oral ibuprofen, oral dipyrone and intramuscular dipyrone. These results are applicable to children with mild to moderate illnesses and a moderately high fever. Further studies are needed to assess the effects of the drugs in children with severe infection or severe malnutrition, and for those children presenting high degrees of fever.

The actual risk of agranulocytosis in patients receiving dipyrone is highly controversial.^12,13^ Media pressure in Brazil recently forced the organizing of a multinational discussion panel. On the basis of the evidence available, this panel concluded that dipyrone should continue to be used in Brazil as an over-the-counter medication.^13–15^ The only way to reach a definitive conclusion on this issue is to do a large study in countries that are heavy users of dipyrone.^13^ A Brazilian study on the epidemiology of aplastic anemia and its risk factors did not show any association between dipyrone use and aplastic anemia.^16^ The LATIN multicenter study for estimating the incidence of aplastic anemia and agranulocytosis in Latin America has calculated a total incidence of 0.5 cases per million individuals per year for agranulocytosis and 2.7 cases per million individuals per year for aplastic anemia.^17^ In a second phase, it will study the correlations with dipyrone.

## CONCLUSIONS

We conclude that the antipyretic efficacy and tolerability are similar for oral ibuprofen, oral dipyrone and intramuscular dipyrone. Oral antipyretics seem more appropriate in feverish children.
